# Effect of β-Carotene on Immunity Function and Tumour Growth in Hepatocellular Carcinoma Rats

**DOI:** 10.3390/molecules17078595

**Published:** 2012-07-18

**Authors:** Bokang Cui, Su Liu, Qibo Wang, Xiaojun Lin

**Affiliations:** Department of Hepatobiliary Oncology, Cancer Center, Sun Yat-sen University, 651 Dongfeng East Road, Guangzhou 510060, Guangdong, China

**Keywords:** β-carotene, immunity function, extraction, ALT, AST

## Abstract

The aim of the present study was to investigate the anticancer and immunity activity of β-carotene in hepatocellular carcinoma (HCC) rats. Three days after transplantation, forty Wistar rats were randomly divided into four groups, each group consisting of 10 animals. These groups were control group (untreated), low-dose β-carotene-treated group (20 mg/kg), middle-dose group (40 mg/kg) and high-dose (60 mg/kg) group. β-Carotene-treated groups were fed with β-carotene (20, 40, 60 mg/kg b.w.) orally for 30 days. Control group was treated with the same volume of physiological saline. Another ten rats were served as the normal group. Results showed that 30 days of β-carotene treatment could significantly inhibit tumour growth, enhance blood NK, IL-2, TNF-α, WBC, TP, ALB and A/G levels, and decrease blood ALT, AST and ALP activities in HCC rats. Pathological analysis of liver tissue showed that β-carotene treatment may decrease damage of liver tissue in HCC rats. It can be concluded that β-carotene may improve the immunity function and inhibit tumour growth in HCC rats.

## 1. Introduction

Hepatocellular carcinoma (HCC) is the fifth most common malignancy in the World. In China, it is the second major cause of cancer death in males and the third in females [1,2]. The pathogenesis of HCC is still poorly understood, and what we know is that some factors are associated with an increased risk of development of the cancer. Patient often have to suffer from cancer that is hard to eradicate. At the present time, most animal studies and clinical trials are being conducted to test a variety of new anticancer strategies [3] such as immunotherapy [4,5], gene therapy [6,7], inhibiting the activity of cancer promoting proteins [8] and inhibiting angiogenesis [9].

β-Carotene, one of the carotenoids, has been thought of value to humans and other species not only as a precursor to vitamin A, but also for having excellent antioxidant properties [10]. Because of the antioxidant properties of β-carotene and other carotenoids, these substances have attracted a great deal of attention over the past two decades as potential chemopreventive agents. Besides being an antioxidant and vitamin A precursor, β-carotene may be more important in our diet than vitamin A because people with low tissue levels of β-carotene were found to be usually prone towards getting a number of different types of cancer [11]. Early epidemiological and animal studies [12,13] advanced the idea that β-carotene can prevent cancer. By the early 1980s, there were a large number of epidemiological studies associating β-carotene intake with lower incidence of epithelial cancers, particularly lung cancer. In 1981 Peto *et al*. [14] summarized the results of prospective and retrospective case-control questionnaire-based studies of populations in eight different countries and provided sufficient evidence of the potential cancer-preventive benefits of β-carotene. However, question as to the safety of the ingestion of high doses of β-carotene have been raised by the Alpha-Tocopherol Beta-Carotene Cancer prevention study (ATBC study) in Finland [15] and another study by the Beta-Carotene and Retinol Efficacy Trial (CARET) [16]. In these both studies a higher incidence of lung cancer among smoking men that received β-carotene was observed in comparison with smokers that did not receive β-carotene. Nevertheless, there are so many epidemiological evidences suggesting the decreased cancer risks with increased consumption of β-carotene [17,18,19,20]. In the present study, we investigate the effect of β-carotene on immunity function and tumour growth in H22 bearing rats.

## 2. Result

Oral administration of β-carotene (20, 40 and 60 mg/kg) significantly decreased the tumour weight and size of the rats in the β-carotene-treated groups in a dose-dependent manner compared to animals to the untreated HCC group. The inhibition rate of β-carotene increased with the increasing concentration (Table 1).

The NK and IL-2 levels in the blood of untreated HCC animals were significantly lower than those of the normal controls. However, there was a significant increase in serum TNF-α in the untreated HCC animals compared to the normal control animals (*p* < 0.01). Administration of β-carotene (20, 40 and 60 mg/kg) dose-dependently significantly increased blood NK, IL-2 and TNF-α of β-carotene-treated HCC animals compared to the untreated HCC rats (Table 2). 

**Table 1:** Effect of β-carotene on tumour weight, size and inhibition rate.

Group	Tumour weight (g)	Tumour size (mm^3^)	Inhibition rate (%)
NC	-	-	-
HCC	0.28 ± 0.02	315.2 ± 19.5	-
HCC + β-carotene (20 mg/kg b.w.)	0.21 ± 0.03 ^c^	291.5 ± 22.4 ^c^	8.3
HCC + β-carotene (40 mg/kg b.w.)	0.16 ± 0.02 ^d^	238.4 ± 15.9 ^d^	23.6
HCC + β-carotene (60 mg/kg b.w.)	0.09 ± 0.01 ^d^	217.1 ± 5.7 ^d^	31.7

^c^ *p* < 0.05, ^d^* p* < 0.01, compared to untreated HCC group; NC; normal control; HCC: hepatocellular carcinoma.

**Table 2:** Effect of β-carotene on NK, IL-2 and TNF-α in rats.

Group	NK (100%)	IL-2 (ng/mL)	TNF-α (ng/mL)
NC	57.21 ± 4.73	4.14 ± 3.28	1.21 ± 0.11
HCC	27.63 ± 2.59 ^b^	1.38 ± 1.53 ^b^	2.05 ± 0.18 ^b^
HCC + β-carotene (20 mg/kg b.w.)	36.82 ± 1.84 ^c^	1.99 ± 1.59	2.22 ± 0.15
HCC + β-carotene (40 mg/kg b.w.)	41.27 ± 3.99 ^d^	2.62 ± 2.22 ^d^	2.39 ± 0.13 ^c^
HCC + β-carotene (60 mg/kg b.w.)	50.52 ± 4.18 ^d^	3.25 ± 2.51 ^d^	2.56 ± 0.11 ^d^

^b^ *p* < 0.01, compared to NC group; ^c^* p* < 0.05, ^d^* p* < 0.01, compared to untreated HCC group.

The peripheral blood WBC, TP, ALB and A/G in un-treated animals were found to decrease significantly compared to the normal controls (*p* < 0.01) (Table 3). Administration of β-carotene (20, 40 and 60 mg/kg) dose-dependently significantly increased peripheral blood WBC, TP, ALB and A/G in β-carotene-treated groups (*p* < 0.01) compared to untreated HCC rats.

**Table 3:** Effect of β-carotene on peripheral blood WBC, TP, ALB and A/G in rats.

Group	WBC (×10^9^/L)	TP (g/L)	ALB (g/L)	A/G
NC	29.06 ± 1.84	70.41 ± 8.34	35.86 ± 4.01	1.83 ± 1.57
HCC	17.94 ± 1.48 ^b^	54.49 ± 3.97 ^b^	31.21 ± 3.29 ^b^	1.21 ± 1.11 ^b^
HCC + β-carotene (20 mg/kg b.w.)	20.14 ± 1.77 ^c^	55.93 ± 6.11	33.71 ± 3.33	1.31 ± 1.21 ^c^
HCC + β-carotene (40 mg/kg b.w.)	22.64 ± 1.83 ^c^	59.17 ± 6.52 ^c^	35.03 ± 2.98 ^c^	1.52 ± 1.44 ^d^
HCC + β-carotene (60 mg/kg b.w.)	24.03 ± 2.06 ^d^	63.11 ± 5.98 ^d^	35.92 ± 3.76 ^c^	1.69 ± 1.63 ^d^

^b^ *p* < 0.01, compared to NC group; ^c^* p *< 0.05, ^d^* p* < 0.01, compared to untreated HCC group.

The blood ALT, AST and ALP activities in untreated animals were found to increase significantly compared to the normal controls (*p* < 0.01) (Table 4). Administration of β-carotene (20, 40 and 60 mg/kg) dose-dependently significantly decreased blood ALT, AST and ALP activities in β-carotene-treated groups (*p* < 0.01) compared to untreated HCC rats.

**Table 4:** Effect of β-carotene on blood ALT, AST and ALP in rats.

Group	ALT (U/L)	AST (U/L)	ALP (U/L)
NC	48.68 ± 4.22	189.53 ± 12.87	77.17 ± 8.63
HCC	92.47 ± 7.83 ^b^	304.15 ± 29.74 ^b^	128.49 ± 13.82 ^b^
HCC + β-carotene (20 mg/kg b.w.)	82.49 ± 7.77 ^c^	287.36 ± 30.11 ^c^	115.39 ± 13.08
HCC + β-carotene (40 mg/kg b.w.)	73.58 ± 7.39 ^d^	254.92 ± 22.86 ^d^	97.47 ± 8.39 ^d^
HCC + β-carotene (60 mg/kg b.w.)	59.73 ± 4.92 ^d^	203.17 ± 23.13 ^d^	80.43 ± 7.29 ^d^

^b^ *p* < 0.01, compared to NC group; ^c^* p* < 0.05, ^d^* p* < 0.01, compared to untreated HCC group.

**Figure 1:**
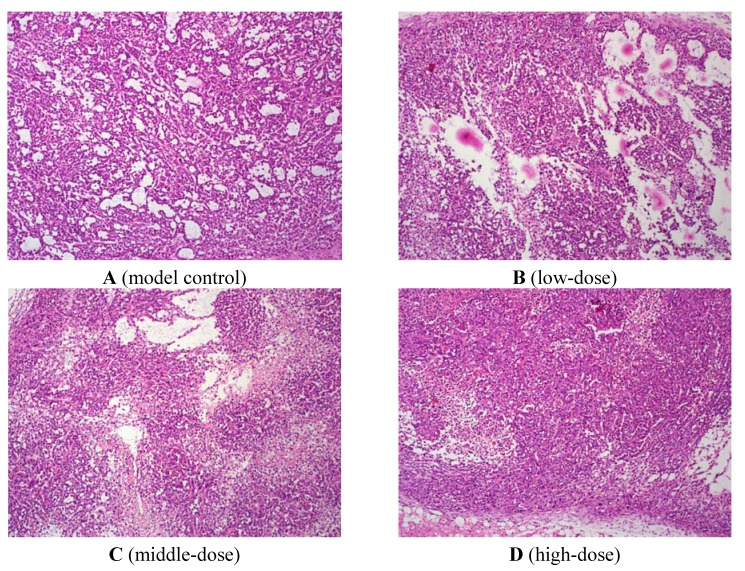
H&E staining of liver.

As shown in Figure 1, normal liver tissue structure in the untreated HCC group disappeared. Tumour cells showed different size, shape (round, oval, irregular shapes). Tumour cells arranged loosely, and cell boundaries were vague. Nucleolus in tumour cells were bigger, and blood sinus in tumour tissue was more abundant. Chromatin was deeply dyed, and cytoplasmic proportion was smaller. Nuclear fission could be observed. In β-carotene-treated group, nuclear fission in tumour cells was less, and lots of lymphocyte and leukocyte infiltration were observed. This indicated that β-carotene could inhibit tumour cell growth.

## 3. Discussion

Hepatocellular carcinoma (HCC) is the most common type of liver cancer, being the fourth leading cause of cancer death worldwide [21]. Development of HCC involves multiple steps including chronic hepatitis, cirrhosis, precancerous lesions and carcinoma [22,23,24]. In the present study, we examined the antitumour activity of β-carotene in HCC rats. Our work confirmed that administration of β-carotene could significantly inhibit tumour cell growth in a dose-dependent manner. 

Natural killer (NK) cells are lymphocytes that have spontaneous and non-MHC-restricted cytotoxic activity directed against a variety of tumor, virus-infected, or allogeneic cell targets. A considerable quantity of accumulated evidence shows that the NK cell plays an important role in the enhancement of host defenses [25,26,27,28,29,30]. Interleukin-2 (IL-2) is an interleukin, a type of cytokine signaling molecule in the immune system. It is a protein that attracts white blood cells (lymphocytes), the cells that are responsible for immunity. It is part of the body’s natural response to microbial infection, and in discriminating between foreign (non-self) and self. IL-2 mediates its effects by binding to IL-2 receptors, which are expressed by lymphocytes. TNF-α is a key factor that contributes to the triggering of an inflammatory cascade involving the induction of cytokines after liver injury [31]. Several studies have demonstrated that some antioxidants significantly diminished TNF-α production [32,33].

In the present study, decreased NK, IL-2 and increased TNF-α were observed in HCC rats after β-carotene treatment. This indicated that β-carotene treatment may increase the capacity of spleen cells inducing IL-2 and peritoneal macrophages inducing TNF-α. It was supposed that the antitumour activity of β-carotene might be closely associated with restoring the body’s immunity function and increasing generation of IL-2, TNF-α etc cytokines.

Disregulation of inflammation and immune system have been implicated in development and progression of cancer [34]. Plasma proteins including WBC were synthesized in liver. When liver cells were damaged, synthesis of plasma proteins was inhibited. Liver is the target organ of toxicity [35], and the leakage of hepatic enzymes such as ALT, AST and ALP are commonly used as an indirect biochemical index of hepatocellular damage [36]. In the present finding, H22-induced liver cancer caused a significant increase in the activities of ALT, AST and ALP, probably resulting from hepatocyte membrane damage. If the liver is injured, its cells spill out the enzymes into blood. These results are consistent with the previous findings realized by some research groups who had found an association between As toxicity and the increased oxidative stress of rats [37,38]. Our work showed that β-carotene treatment may significantly enhance TP, ALB, A/G level, and decrease ALP, ALT, AST level. As a result, these improved tumor-bearing rats’ liver function. Finally, pathological analysis of liver tissue showed that β-carotene treatment may decrease damage of liver tissue in HCC rats.

## 4. Experimental

### 4.1. Rats and Cell Lines

H22 cells (murine hepatocarcinoma cells), purchased from the Shanghai Institute for Biological Sciences (Chinese Academy of Sciences, Shanghai, China), were cultured in RPMI-1640 containing 10% heat-inactivated fetal bovine serum, 100 units/mL penicillin G, 100 mg/mL streptomycin, and 2 mmol/L glutamine. The cocultures of H22 cells were then freeze-thawing 5 times to inactivate living H22 hepatocarcinoma cells for following studies.

### 4.2. Animal Grouping and Treating

1 × 10^7^ /mL of H22 cells were injected into the right oxter of each rat. Then, forty Wistar rats (220–250 g) were randomly divided into four groups, each group consisting of 10 animals. They were control group (untreated) (HCC), low-dose group [HCC + β-carotene (20 mg/kg b.w.)], middle-dose group [HCC + β-carotene (40 mg/kg b.w.)] and high-dose group [HCC + β-carotene (60 mg/kg b.w.)] β-carotene-treated group. β-Carotene was dissolved in soybean oil. β-Carotene-treated groups were fed with β-carotene oil solution [20, 40, 60 mg β-carotene/kg b.w.; human equivalent dose (HED) 3.2, 6.5, 9.7 mg/kg] orally for 30 days. Model control group (HCC) was treated with the same volume of vehicle (soybean oil). Another ten rats were served as the normal control group (NC). Twenty-four hours later after the last drug administration, the animals were euthanized. Blood samples were collected and centrifuged at 3,000 r/min at 4 °C for 10 min to obtain serum. Tumor tissue was totally excised from the animal and accurately weighed. Meanwhile, the livers were excised from the animal and stored at −80 °C. The inhibition ratio (IR) of the tumor growth was calculated by the following equation: IR (%) = 100 × (A − B)/A, where A is the average tumor weight of the control group and B is the average tumor weight of the treated group.

### 4.3. Biochemical Analysis

The activity of natural killer (NK) cells was assayed as described previously (Reynolds and Herberman, [39]). Single cell suspensions were adjusted to six concentrations: 2 × 10^7^, 1 × 10^7^, 5 × 10^6^, 2.5 × 10^6^, 1.25 × 10^6^, and 0.625 × 10^6^ cells/mL. The target cells, YAC-1 cells, were labeled with 51Cr and added to each well of a 96-well plate in a volume of 0.1 mL. The effector cells (0.1 mL) were added to each of two replicate wells of target cells at each effector concentration to obtain effector/target (E:T) ratios of 200:1, 100:1, 50:1, 25:1, 12.5:1, and 6.25:1. The spontaneous release and the maximum release were determined by adding 0.1 ml of medium and Triton X-100 (0.1%) to each of 12 replicate wells containing the target cells, respectively. Following a 4-h incubation, the plates were centrifuged, and 0.1 mL of the supernatant was removed from each well and counted. The mean percentage of cytotoxicity at each effector concentration was determined. IL-2, TNF-α, AST, ALT, and ALP levels were measured with commercially available kits. All procedures followed the manufacturers’ instructions. White blood cell (WBC) count were measured using a hematological autoanalyzer (Advia 120E, Bayer, Newbury, UK). Liver function tests (TP, ALB, A/G), liver histology, portal blood flow and portal pressure measured by MRF-1200 electromagnetic flowmetry (Nikon, Tokyo, Japan).

### 4.4. Histological Evaluation

Histological assessment was used to complete the study of liver damage. For this purpose, each liver tissue was fixed in 10% buffered-neutral formalin, routinely processed, embedded in paraffin and sections of 5 mm thick were cut. Hematoxylin and eosin (H&E) were used for staining. The sections were analyzed by a certified pathologist ignoring the sample assignments to experimental groups. A minimum of three fields of each liver slide was morphologically evaluated.

### 4.5. Statistical Analysis

Statistical analyses were performed using SPSS 10.0 for Windows (SPSS Inc., Chicago, IL, USA). Data are expressed as Mean ± SD. One-way ANOVA was preformed first, and if significant, then the group mean differences was determined by a post-hoc test such as Scheffe’s test or LSD. Statistical significance was considered at *p* < 0.05.

## 5. Conclusion

β-Carotene may improve immunity function and inhibit tumour growth in HCC rats.
